# Predicting feeding-tube dependence in patients following endotracheal extubation: a two-item swallowing screen

**DOI:** 10.1186/s12890-021-01771-5

**Published:** 2021-12-06

**Authors:** Shu-Fen Siao, Wen-Hsuan Tseng, Tyng-Guey Wang, Yu-Chung Wei, Tzu-Yu Hsiao, Shih-Chi Ku, Cheryl Chia-Hui Chen

**Affiliations:** 1 Department of Nursing, National Taiwan University College of Medicine, Taipei, Taiwan R.O.C.; 2 Department of Otolaryngology, National Taiwan University Hospital and National Taiwan University College of Medicine, Taipei, Taiwan R.O.C.; 3 Department of Physical Medicine and Rehabilitation, National Taiwan University Hospital and National Taiwan University College of Medicine, Taipei, Taiwan R.O.C.; 4 Graduate Institute of Statistics and Information Science, National Changhua University of Education, Changhua, Taiwan R.O.C.; 5 Department of Internal Medicine, National Taiwan University Hospital and National Taiwan University College of Medicine, 7, Chung Shan S. Rd., Zhongzheng Dist., Taipei, 100 Taiwan R.O.C.; 6 Department of Nursing, National Taiwan University Hospital and National Taiwan University College of Medicine, Taipei, Taiwan R.O.C.

**Keywords:** Endotracheal intubation, Dysphagia, Deglutition, Swallowing, Feeding-tube dependence, Intensive care unit, Nutritional status

## Abstract

**Background:**

To meet the surging demands for intubation and invasive ventilation as more COVID-19 patients begin their recovery, clinicians are challenged to find an ultra-brief and minimally invasive screen for postextubation dysphagia predicting feeding-tube dependence persisting for 72 h after extubation.

**Methods:**

This study examined the predictive validity of a two-item swallowing screen on feeding-tube dependence over 72 h in patients following endotracheal extubation. Intensive-care-unit (ICU) patients (≥ 20 years) successfully extubated after ≥ 48 h endotracheal intubation were screened by trained nurses using the swallowing screen (comprising oral stereognosis and cough-reflex tests) 24 h postextubation. Feeding-tube dependence persisting for 72 h postextubation was abstracted from the medical record by an independent rater. To verify the results and cross-check whether the screen predicted penetration and/or aspiration during fiberoptic endoscopic evaluation of swallowing (FEES), participants agreeing to receive FEES were analyzed within 30 min of screening.

**Results:**

The results showed that 95/123 participants (77.2%) failed the screen, which predicted ICU patients’ prolonged (> 72 h) feeding-tube dependence, yielding sensitivity of 0.83, specificity of 0.35, and accuracy of 0.68. Failed-screen participants had 2.96-fold higher odds of feeding-tube dependence (95% CI, 1.13–7.76). For the 38 participants receiving FEES, the swallowing screen had 0.89 sensitivity to detect feeding-tube dependence and 0.86 sensitivity to predict penetration/aspiration, although specificity had room for improvement (0.36 and 0.21, respectively).

**Conclusion:**

This ultra-brief swallowing screen is sufficiently sensitive to identify high-risk patients for feeding-tube dependence persisting over 72 h after extubation. Once identified, a further assessment and care are indicated to ensure the prompt return of patients’ oral feeding.

**Trial registration:**

NCT03284892, registered on September 15, 2017.

**Supplementary Information:**

The online version contains supplementary material available at 10.1186/s12890-021-01771-5.

## Introduction

Identifying critically ill patients at risk for swallowing dysfunction is a priority after extubation. Postextubation dysphagia (PED) observed in previously dysphagia-naïve intensive care unit (ICU) patients is well recognized as a common sequela of intubation and can have serious consequences, including feeding-tube dependence [1], increased risk of pneumonia [2–4], malnutrition [5], prolonged lengths of ICU and hospital stays [2, 6], and higher 90-day mortality [6]. Although many swallowing-screen tools are available, e.g., screens for dysphagia or aspiration, very few have been studied in ICU patients [7].

PED prevalence is, therefore, highly variable, ranging between 3 and 93% [1]. This wide range is likely exacerbated by heterogeneity in study design, including diagnostic and screening methods. In theory, PED screening comprises two approaches: screening for dysphagia or screening for risk of aspiration. At the screening level, distinguishing between dysphagia (physiologic swallowing dysfunction) and risk of aspiration (a result of physiologic swallowing dysfunction or dysphagia) is important. All three currently available PED screens (a bedside swallowing evaluation [administered only by speech therapists] [8], the Yale Swallow Protocol [9], and Postextubation Dysphagia Screening [10]) target aspiration risk and include a 3-oz water-swallow test.

However, these screening tools are not yet consistently applied clinically [11], resulting in a theory–practice gap. This underuse of screening tools is due to several reasons, including lack of guidelines to recommend their use, uncertainty about what to do with the screening results, and lack of time and specialized trained personnel to administer the screens [12–15]. In many cases, nurses are responsible for administering these screens, i.e., introducing liquids or foods that result in clinical signs of aspiration such as coughing, choking, and wet vocal quality that usually indicate PED [16, 17]. Although the resulting clinical signs are seemingly straightforward, many nurses do not feel confident in judging them [12], especially when it has been suggested that 25% of patients aspirate silently [18]. On the other hand, nurses are concerned that aspiration triggered by water swallow tests can harm frail ICU patients even further [15].

The most efficient approach for consistently identifying cases of dysphagia may be a two-step approach in which a sensitive, user-friendly, and ultra-brief screen is administered, followed by a swallow assessment in those who “fail” the screen. Moreover, a screening tool for dysphagia that focuses more on physiologic swallow dysfunction is not yet available. Thus, the current study was designed to examine a swallowing screen that targets dysphagia and physiologic swallow dysfunction and includes tasks that are nurse-friendly and aspiration-risk free. We hypothesized that a two-item swallowing screen consisting of oral stereognosis (ability to identify shapes in the mouth only by touch, indicating oral functioning and/or cognitive ability to respond) and cough reflex (indicating airway defense) tests would predict patients with feeding-tube dependence persisting for 72 h following endotracheal extubation, a proxy for delayed return to oral feeding. By asking patients to identify three shapes (square, star, and round) of standardized acrylic resin as lollipop-like objects by oral manipulation (without seeing) and observing whether they had cough responses via citric acid inhalation using nebulizer masks, nurses could administer oral stereognosis and cough reflex tests at bedside.

As an exploratory outcome, predicted risk of aspiration was also cross-checked by the swallowing screen. Participants were offered the option of receiving the gold-standard fiberoptic endoscopic evaluation of swallowing (FEES) performed by a trained otolaryngologist. We hypothesized that participants who failed the screen were more likely to have penetration and/or aspiration during FEES. The screen and FEES were performed within 30 min of each other using standardized protocols [19].

## Methods

### Study design, settings, and participants

**Fig. 1:**
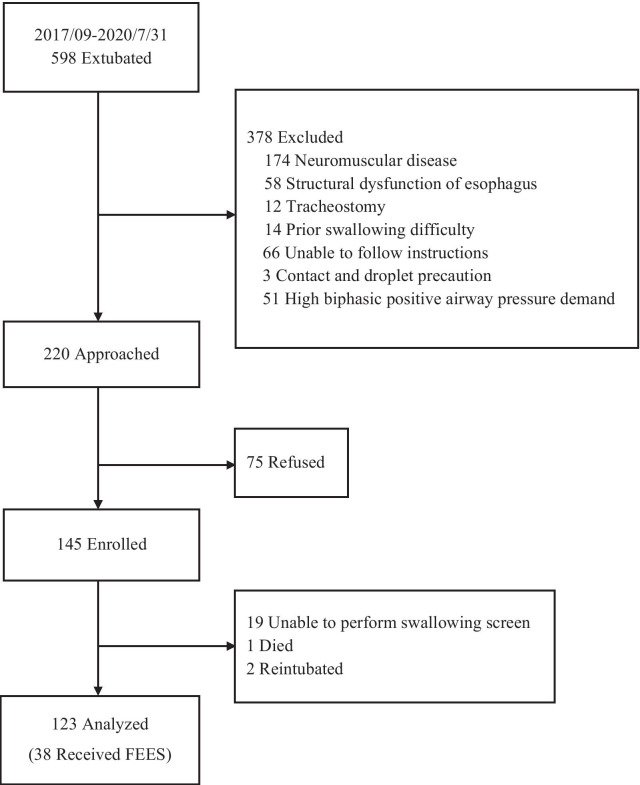
Study flow diagram

Between September 2017 and July 2020, participants were enrolled from a tertiary medical center in Taipei, Taiwan. Consecutive patients (≥ 20 years old) admitted to the medical center’s six medical ICUs were recruited if they had received emergency oral endotracheal intubation for at least 48 h and were successfully extubated. Patients were excluded if they (1) had a history of neuromuscular disease (e.g., parkinsonism or stroke) or head and neck deformities, (2) had a preexisting swallowing difficulty, (3) received tracheostomy, (4) could not follow verbal instructions, or (5) were placed on contact and droplet precaution (e.g., for open tuberculosis) or had noninvasive positive pressure ventilators (NPPV) continuously after extubation. The study was approved by the Human Research Ethics Committee of the study hospital and registered at the Clinical Trials Registry (Trial No: NCT03284892, [15/09/2017]). Written informed consent was signed by participants or their surrogates. The detailed study flow is in Fig. 1.

### Data collection and outcome measures

Data on participant characteristics (age, sex, current smoker [yes/no], body weight, body mass index), ICU admission diagnosis (respiratory failure, cardiac emergency, noncardiogenic shock, and other), Charlson comorbidity index (CCI), illness severity (Acute Physiological and Chronic Health Evaluation II; APACHE II), size of endotracheal tube, and lengths of intubation and ICU stay were abstracted from medical records. Baseline data on increased respiratory rate (≥ 25 breaths/min; yes/no), high suctioning demand (over 6 per day; yes/no), oxygen demand (room air, nasal cannula, simple mask, nonrebreathing mask, or NPPV), and nothing by mouth status (yes/no) were evaluated by a research nurse after extubation.

#### Swallowing screen

All participants were evaluated 24 to 48 h after endotracheal extubation by trained nurses using a standardized swallowing screen (consisting of oral stereognosis and cough reflex). Participants with intact oral stereognosis and cough reflex were defined as having “passed” the screen, while all other participant responses were defined as having “failed.” Specifically, oral stereognosis was tested by a set of three standardized researcher-made, acrylic resin, lollipop-style test pieces (square, star, and round “lollipop” shapes) based on prior studies [20–22]. Participants were given 30 s (s) to visually inspect these three test pieces along with a diagram of their corresponding shapes before testing began. The trained nurse then inserted these test pieces, one-by-one, randomly, placing it on the dorsum of participants’ tongue, allowed the participant 10 s to manipulate the test piece in the mouth (participants or research nurses held the 3.5″ lollipop stick to ensure safety), and participants had another 10 s to identify the object on the corresponding diagram. Participants who correctly identified all three shapes were considered to have intact oral stereognosis.

Cough reflex, based on European Respiratory Society guidelines, was tested via up to three trials of citric acid inhalation to prevent tachyphylaxis [23, 24], i.e., a rapid and short-term onset of drug tolerance. In this study, participants were kept in a sitting position (at least 60 degrees) and asked to breathe normally. Via an oxygen-driven nebulizer mask, 0.4 mol/L citric acid was administered in up to three trials, and participants’ cough responses were observed for 15 s. Participants who had cough responses in at least two trials were coded as having an intact cough reflex. Given that coughing emits aerosol droplets, trained nurses took appropriate precautionary isolation steps to avoid disease transmission.

#### Outcome: feeding-tube dependence persisting 72 h after extubation

As the primary outcome variable, feeding-tube dependence persisting 72 h after extubation (yes/no), was abstracted from electronic medical records by an independent rater who was blinded to the screen results. We also examined penetration and/or aspiration using FEES by a board-certified otolaryngologist. Subgroup participants who opted to receive FEES within 30 min of completing the screen were assessed with a standardized set of liquid dye and solid puree trials [19, 25]. Under FEES, penetration and/or aspiration was defined as the entry of food (either liquid dye or solid puree) into the airway (laryngeal vestibule, vocal folds, and trachea) immediately post-swallowing.

### Statistical analysis

All analyses were performed with SAS Statistical software version 9.4 (SAS Institute Inc., Cary, NC). Significance was set at *P* < 0.05. Continuous variables were described by the median and interquartile range. Categorical variables were described by numbers with percentages. Pass and failure rates on the swallowing screening were reported and tabulated with outcome variables. Risk of feeding-tube dependence in the failed screen cohort was estimated using logistic regression and adjusted for relevant confounders, including length of intubation (days) and CCI score, to obtain adjusted odds ratios (ORs) (95% confidence interval [95% CI]). Sensitivity, specificity, and accuracy were calculated to reflect the swallowing screen’s diagnostic accuracy. Sensitivity analyses separating the oral stereognosis and cough reflex tests were also conducted to examine their individual performance and item difficulty.

## Results

Of the 145 enrolled participants, 123 completed both the screening and oral intake follow-ups, with 22 excluded due to reintubation (n = 2), death (n = 1), and inability to complete screens due to delirium status (n = 19). For subgroup analysis, 38 participants completed FEES within 30 min of the screen. Participants who opted to receive FEES (n = 38) and those who declined (n = 85) were not different in terms of age (*P* = 0.58), gender (*P* = 0.85), CCI score (*P* = 0.15), APACHE II (*P* = 0.67), and length of intubation (*P* = 0.43). For details of the study flow, see Fig. 1.

Participants’ demographic and clinical characteristics (N = 123) are in Table 1. Participants’ median age was 67 years (IQR = 54–74), with 40.7% being female. The majority of participants’ endotracheal tube sizes were 7.0 Fr. and 7.5 Fr., with a median 6 days (IQR = 4.2–10.8) of intubation. The median length of ICU stay was 10 days (IQR = 7–16). After extubation, 10.6% had increased respiratory rate (≥ 25 breaths/min), 49.6% had high suctioning demand requiring more than six suctions per day, and all had oxygen demand (82.1% received simple mask therapy, and 10.6% received nonrebreathing mask or NPPV therapy).

**Table 1:** Participant characteristics (N = 123)

Characteristic	Total
Age, years, median (IQR)	67 (54–74)
Female, n (%)	50 (40.7)
Current smoker, n (%)	20 (16.3)
Body mass index, median (IQR)	23.2 (20.4–26.4)
Charlson Comorbidity Index, median (IQR)	2 (1–4)
*ICU admission diagnosis, n (%)*	
Respiratory failure	66 (53.7)
Cardiac emergency	25 (20.3)
Noncardiogenic shock	27 (21.9)
Other	5 (4.1)
APACHE II at ICU admission, median (IQR)	20 (17–26)
*APACHE II at ICU admission, n (%)*	
0–24	84 (68.3)
> 25	39 (31.7)
*Endotracheal tube size (Fr), n (%)*	
6.5	1 (0.8)
7.0	53 (43.1)
7.5	68 (55.3)
8.0	1 (0.8)
Length of intubation (d), median (IQR)	6 (4.2–10.8)
*Postextubation baseline*	
Length of ICU stay (d), median (IQR)	10 (7–16)
Increased respiratory rate (≥ 25 breaths/min), n (%)	13 (10.6)
High suctioning demand, n (%)	61 (49.6)
*Oxygen demand, n (%)*	
Room air	0 (0)
Nasal cannula	9 (7.3)
Simple mask	101 (82.1)
NRM or NPPV	13 (10.6)
Nothing by mouth, n (%)	123 (100)

Abbreviations: IQR, interquartile range; NRM, nonrebreathing mask; APACHE II: Acute Physiological and Chronic Health Evaluation II; CCI: Charlson comorbidity index; Fr: French gauge system; ICU: Intensive care unit; NPPV: Noninvasive positive pressure ventilator

### Feeding-Tube dependence versus failed screen

Of 123 participants, 28 (22.8%) passed the screen (intact oral stereognosis and cough reflex), and 95 (77.2%) failed. By 72 h postextubation, 83 participants (67.4%) remained feeding-tube dependent and had delayed return to oral feeding, yielding a sensitivity of 0.83 (95% CI, 0.75–0.91), specificity of 0.35 (95% CI, 0.20–0.49), and accuracy of 0.68 (95% CI, 0.59–0.75) (Table 2). Logistic regression models revealed that belonging to the failed screen group was associated with 2.96-fold higher odds of feeding-tube dependence (95% CI, 1.13–7.76; *P* = 0.027), after adjusting for relevant confounders (length of intubation and CCI score) (Table 3).

**Table 2:** Swallowing screen to predict prolonged feeding-tube dependence (N = 123)

	Feeding-tube dependence		Total
	Yes ( +)	No ( −)	
*Swallowing screen*^*a*^			
Failed, n	69	26	95
Passed, n	14	14	28
Total	83	40	123

^a^Sensitivity (95% CI) = 0.83 (0.75–0.91), Specificity (95% CI) = 0.35 (0.20–0.49), Accuracy (95% CI) = 0.68 (0.59–0.75)

**Table 3:** Risk of failed screen cohort on prolonged feeding-tube dependence

	Feeding-tube dependence			
	OR (95% CI)	*P* value	AOR (95% CI)	*P* value
*Failed screen*^*a*^	2.65 (1.11–6.31)	0.027	2.96 (1.13–7.76)	0.027
Length of intubation, days	–		1.15 (1.05–1.28)	0.005
Charlson comorbidity index	–		1.24 (1.02–1.51)	0.031

Abbreviations: OR, odds ratio; AOR, adjusted odds ratio

^a^Passed group as reference

### Subgroup analysis

Among 38 participants who opted to receive FEES, 36.8% (n = 14) had penetration and/or aspiration immediately after swallowing. To predict feeding-tube dependence, the swallowing screen performed slightly better in this subgroup and yielded sensitivity of 0.89, specificity of 0.36, and accuracy of 0.74. For penetration and/or aspiration, high sensitivity (0.86) was achieved, but specificity (0.21), and accuracy (0.45) were less than optimal (Table 4).

**Table 4:** Subgroup analysis of participants who opted to receive FEES (n = 38)

	Yes (+)	No (−)	Total	
*Feeding-tube dependence*				
Swallowing screen				
Failed, n	24	7	31	Sensitivity = 0.89
Passed, n	3	4	7	Specificity = 0.36
Total	27	11	38	Accuracy = 0.74
*Aspiration/penetration*				
Swallowing screen				
Failed, n	12	19	31	Sensitivity = 0.86
Passed, n	2	5	7	Specificity = 0.21
Total	14	24	38	Accuracy = 0.45

### Sensitivity analysis in item performance

We then separated the two components of the screen, the oral stereognosis and cough reflex tests, to examine their individual performance. Oral stereognosis itself has an accuracy (0.65) comparable to that of the two-item screen (0.68), while the cough reflex accuracy is 0.47. Combining the two indeed improves screen performance (Additional file 1: Table S1).

Nevertheless, this two-item screen identified 26 false-positive cases that required further swallowing assessment, along with 14 false-negative cases that would have been missed. We further examined item difficulty comparing these 26 cases with true-positive (n = 69) and false-negative cases (n = 14) in their performances (Additional file 1: Table S2). Comparing only 7.3% of true positives and 7.7% of false positives that correctly identified three shapes in oral stereognosis, high rates of participants (63.8% of true positives and 73.1% of false positives) had two reflexive cough responses, suggesting a ceiling effect in the cough reflex test.

Moreover, our review of the medical records of 14 false-negative cases showed that 50% of these false-positive cases with prolonged feeding-tube dependence were due to medical conditions; seven had an NPO order (due to gastrointestinal bleeding, n = 3; colon perforation, n = 2; and stridor/desaturation, n = 2) and two had a tube-feeding order due to severe underweight (body mass index < 18) requiring supplements to boost energy intake.

## Discussion

The most important finding of this study was that our swallowing screen, consisting of oral stereognosis and cough reflex tests, were sufficiently sensitive to identify postextubation patients with feeding-tube dependence that persisted 72 h and beyond, despite a 0.35 specificity. Although an overall accuracy of 0.68 leaves room for improvement and false positives may bring diagnostic and care burden, casting a wider net in the first stage of case-finding may enable nurses and physicians to triage patients needing further assessment before and during their recovery to oral feeding.

Three study findings warrant emphasis. **First**, the sufficient accuracy of our screen, supports, at least partly, the pathophysiological mechanism of impaired oral stereognostic ability and cough reflex affecting oral feeding after extubation. Although oral stereognosis has not been well-studied, it was impaired after extubation when compared to an aged-matched, non-ICU patient group [21]. In this study, we established the temporal relation between impaired oral stereognosis and feeding-tube dependence by showing that the former predicted the latter. Studies are warranted to investigate the roles of oral stereognosis in feeding-tube dependence, both in terms of test properties and magnitude of impact, to refine the screen items.

For the cough reflex, having two reflexive cough responses to 0.4% citric acid alone is highly specific (0.83), consistent with a previous study [23], to rule out feeding-tube dependence in this critically ill group. Sensitivity, however, was only 0.30 (more false negatives); thus, using this item alone is not acceptable. For improvement, ceiling effects found in our sensitivity analysis provide a direction (i.e., increasing cough counts to pass or adding cough strength to raise the ceiling). Moreover, the cough reflex test is yet to be standardized [26], and further studies could consider standardizing the protocols while titrating the passing threshold to enhance its testing performance.

**Second,** the low specificity of our two-item screen may come with more false positives, which might add diagnostic burden to staff as more cases are identified and referred. However, given the purpose of dysphagia screening, high sensitivity is preferred not to miss potential cases. As such, false negatives (i.e., those cases of feeding tube dependence are missed), are noteworthy. After careful review, we found that 50% of those false negatives had NPO or tube-feeding orders due to their medical conditions such as gastrointestinal bleeding or severe underweight requiring extra intake energy. This finding not only highlights the complexity of conducting studies in critically ill patients but also alleviates concerns about important cases missed by our screening. Whether systematically using this screen as a two-step approach, in which a further assessment follows “failed” cases, improves patients’ outcomes requires an impact-evaluation study.

**Third,** subgroup analysis of 38 participants receiving FEES indicated that this screen performed better in predicting feeding-tube dependence than penetration and/or aspiration during FEES (0.74 vs. 0.45 accuracy, respectively). This difference at least partially supports our aim to screen for dysphagia, rather than aspiration. The three currently available PED screens all focus on aspiration and utilize a 3-oz water swallowing test [7], whereas growing data suggest that not all nurses are confident in performing these screens, correctly identifying signs of aspiration (i.e., wet voice). One recent study revealed that 81% of nurses working in the cardiac ICU reported having no formal training in swallow screening [12]. Nearly half, 48%, of ICU nurses reported feeling “somewhat” or “not” confident in performing screens [12]. Our two-item swallowing screen provides important alternatives. Indeed, in the era of the Covid-19 pandemic, if needed, nurses could remotely test for oral stereognosis to avoid unnecessary contact; for just this item alone, accuracy was 0.65. To increase screen accuracy to 0.68, cough reflex could be added but adequate personal protection equipment and physical distancing are required to prevent disease transmission. For patients who fail the screen, a further swallow assessment is indicated to enhance the return of their oral feeding and maximize patient outcomes.

## Limitations

This study had important limitations. **First,** the swallowing screen is a two-item tool designed to be administered by nurses at the ICU bedside with minimal respondent burden. The capacity to enhance diagnostic accuracy is limited, for example by modifying criteria or cutoffs, as we decided beforehand that participants would pass the screen if they identified all three shapes and had two cough responses. **Second,** although our primary endpoint, feeding-tube dependence persisting over 72 h after extubation, often reflects physiologic swallowing dysfunction, it may also reflect conditions other than swallowing (i.e., gastrointestinal disease, nutritional conditions, or even practice patterns). As such, whether our screen has added value in other ICU settings remains to be tested. **Third**, our sample was not large enough (especially with only 38 participants receiving FEES) to allow more complex confirmatory analyses. Our results, therefore, need to be interpreted cautiously and replicated in other studies with larger samples. **Fourth,** our study was limited by enrolling participants from a single institution with an enrollment rate of 65.9% (145 of 220 invited patients) and completion rate of 84.8% (N = 123 of 145 enrolled; 19 unable to complete the screen, 1 death and 2 reintubations). While all eligible patients were offered enrollment, selection bias remains a limitation that will need to be addressed by replicating the findings in future studies.

## Conclusions

Postextubation dysphagia observed in previously dysphagia-naïve ICU patients is associated with poor outcomes. An essential beginning to their care is adequate screening for dysphagia. Our ultra-brief swallowing screen, comprising oral stereognosis and cough reflex tests, is sufficiently sensitive to identify patients at high risk for feeding-tube dependence persisting over 72 h after extubation. Once identified, further assessment and care are indicated to ensure the prompt return of patients’ oral feeding.

## Supplementary Information

**Additional file 1:** Sensitivity analysis in item performance.

## Abbreviations

95% CI: 95% Confidence interval
ORs: Odds ratios
APACHE II: Acute Physiological and Chronic Health Evaluation II
CCI: Charlson comorbidity index
FEES: Fiberoptic endoscopic evaluation of swallowing
ICU: Intensive care unit
NPPV: Noninvasive positive pressure ventilator
PED: Postextubation dysphagia

## Declarations

### Availability of data and material

The datasets used and/or analyzed during the present study are available from the corresponding author on reasonable request.
