# Supplementary material for: Predicting feeding-tube dependence in patients following endotracheal extubation: a two-item swallowing screen
Source: BMC Pulm Med. 2021 Dec 6;21:403. doi: 10.1186/s12890-021-01771-5 (PMC8647059; doi:10.1186/s12890-021-01771-5)
Supplement: Supplementary file 1 — Additional file 1: Sensitivity analysis in item performance. [file 12890_2021_1771_MOESM1_ESM.docx]

**Table S1** 2x2 contingency table of individual oral stereognosis vs. cough reflex tests

|  | Feeding-Tube Dependence  (n=123) | |  | Aspiration/Penetration  (n=38) | |
| --- | --- | --- | --- | --- | --- |
|  | Yes (＋) | No (－) |  | Yes (＋) | No (－) |
| **Oral stereognosis test** |  |  |  |  |  |
| Failed, n | 64 | 24 |  | 12 | 19 |
| Passed, n | 19 | 16 |  | 2 | 5 |
| **Cough reflex test** |  |  |  |  |  |
| Failed, n | 25 | 7 |  | 1 | 6 |
| Passed, n | 58 | 33 |  | 13 | 18 |

Accuracy of oral stereognosis in predicting feeding-tube dependence = 0.65

Accuracy of oral stereognosis in predicting aspiration/penetration during FEES = 0.45

Accuracy of cough reflex in predicting feeding-tube dependence = 0.47

Accuracy of cough reflex in predicting aspiration/penetration during FEES = 0.50

**Table S2** Item difficulty in oral stereognosis and cough reflex tests

|  | True positive  (n=69) | False positive  (n=26) | False negative  (n=14) |
| --- | --- | --- | --- |
| **Oral stereognosis test**, n (%) |  |  |  |
| Failed |  |  |  |
| 0 piece^*^ | 11 (15.9) | 3 (11.5) | 0 |
| 1 piece | 33 (47.8) | 8 (30.8) | 0 |
| 2 pieces | 20 (29) | 13 (50) | 0 |
| Passed |  |  |  |
| 3 pieces | 5 (7.3) | 2 (7.7) | 14 (100) |
| **Cough reflex test**, n (%) |  |  |  |
| Failed |  |  |  |
| 0 cough | 18 (26.1) | 4 (15.4) | 0 |
| 1 cough | 4 (15.4) | 3 (11.5) | 0 |
| Passed |  |  |  |
| 2 coughs | 44 (63.8) | 19 (73.1) | 14 (100) |

^*^ Piece refers to each variously shaped object placed on participants’ tongue
